# Small LEA proteins mitigate air-water interface damage to fragile cryo-EM samples during plunge freezing

**DOI:** 10.1063/4.0000830

**Published:** 2025-10-27

**Authors:** Kaitlyn M Abe, Gan Li, Qixiang He, Timothy Grant, Ci Ji Lim

**Affiliations:** 1 University of Wisconsin-Madison, Madison, WI, USA; 2 Morgridge Institute for Research, Madison, WI, USA

## Abstract

Air-water interface (AWI) interactions during cryo-electron microscopy (cryo-EM) sample preparation cause significant sample loss, hindering structural biology research. Organisms like nematodes and tardigrades produce Late Embryogenesis Abundant (LEA) proteins to withstand desiccation stress. Here we show that these LEA proteins, when used as additives during plunge freezing, effectively mitigate AWI damage to fragile multi-subunit molecular samples. The resulting high-resolution cryo-EM maps are comparable to or better than those obtained using existing AWI damage mitigation methods. Cryogenic electron tomography reveals that particles are localized at specific interfaces, suggesting LEA proteins form a barrier at the AWI. This interaction may explain the observed sample-dependent preferred orientation of particles. In summary, we show LEA proteins can offer a simple, cost-effective, and adaptable approach for cryo-EM structural biologists to overcome AWI- related sample damage, potentially revitalizing challenging projects and advancing the field of structural biology.

